# Pollen nightmare: elevated airborne pollen levels at night

**DOI:** 10.1007/s10453-016-9441-7

**Published:** 2016-05-10

**Authors:** Ł. Grewling, P. Bogawski, M. Smith

**Affiliations:** Laboratory of Aeropalynology, Faculty of Biology, Adam Mickiewicz University, Umultowska 89, 61-614 Poznan, Poland

High airborne pollen concentrations are generally associated with daylight hours when it is sunny and warm and plants release pollen into the air (Alcázar et al. 1999; Dahl et al. 2013). In contrast, cooler night-time periods are usually considered to be the time of low-allergy risk. This opinion is often reflected in pollen allergy avoidance strategies presented by the media, where the most commonly repeated recommendation is to stay indoors during the day and plan outdoor activities for the evening. However, there is evidence to suggest that elevated concentrations of airborne pollen might also occur during the evening (e.g. Norris-Hill and Emberlin 1991). So, is the night really a time of low-allergy risk? We present the results of the comparative analysis of pollen concentrations during daytime and night-time hours for five allergenic pollen types (Burbach et al. 2009), i.e. alder (*Alnus* sp.), birch (*Betula* sp.), grasses (Poaceae), mugwort (*Artemisia* sp.) and ragweed (*Ambrosia* sp.).

Airborne pollen grains were collected by volumetric trap (Hirst 1952) in Poznań, Poland (1996–2013). The trap was sited on the roof at the height of 33 m, approximately 1 km south-west of the city centre (52°24′N 16°53′E). Two pollen-counting methods have been applied. From 1996 to 1999 pollen data were counted along twelve vertical transects, while from 2000 to 2013 along four horizontal transects. Both counting methods have been shown to produce comparable results and are recommended by the European Aerobiology Society (Galán et al. 2014). The following time intervals were selected to reflect airborne pollen levels during night and day: 08:00–20:00 (equivalent of daytime, 12 h) and 20:00–08:00 (i.e. night-time, 12 h). This division was made to distinguish the time period that is not generally considered hazardous for allergy patients (from late evening to early morning). The rejection of low concentrations of atmospheric pollen guarantees more robust data (Buters et al. 2012), and so only 24-h periods (from 08:00 to 08:00 next day) with mean pollen levels ≥15 pollen/m^3^ were selected for analysis (*n* = 2177). The bi-hourly pollen concentrations recorded during selected days were averaged for 12-h periods to get mean concentrations for day and night. Daytime and night-time airborne pollen concentrations (mean and maximum values) were compared using the nonparametric Mann–Whitney *U* test (Real Statistics Add-in to Excel). In addition, the frequency (%) of 24-h periods with mean and maximum pollen levels higher at night has been calculated.

**Fig. 1:**
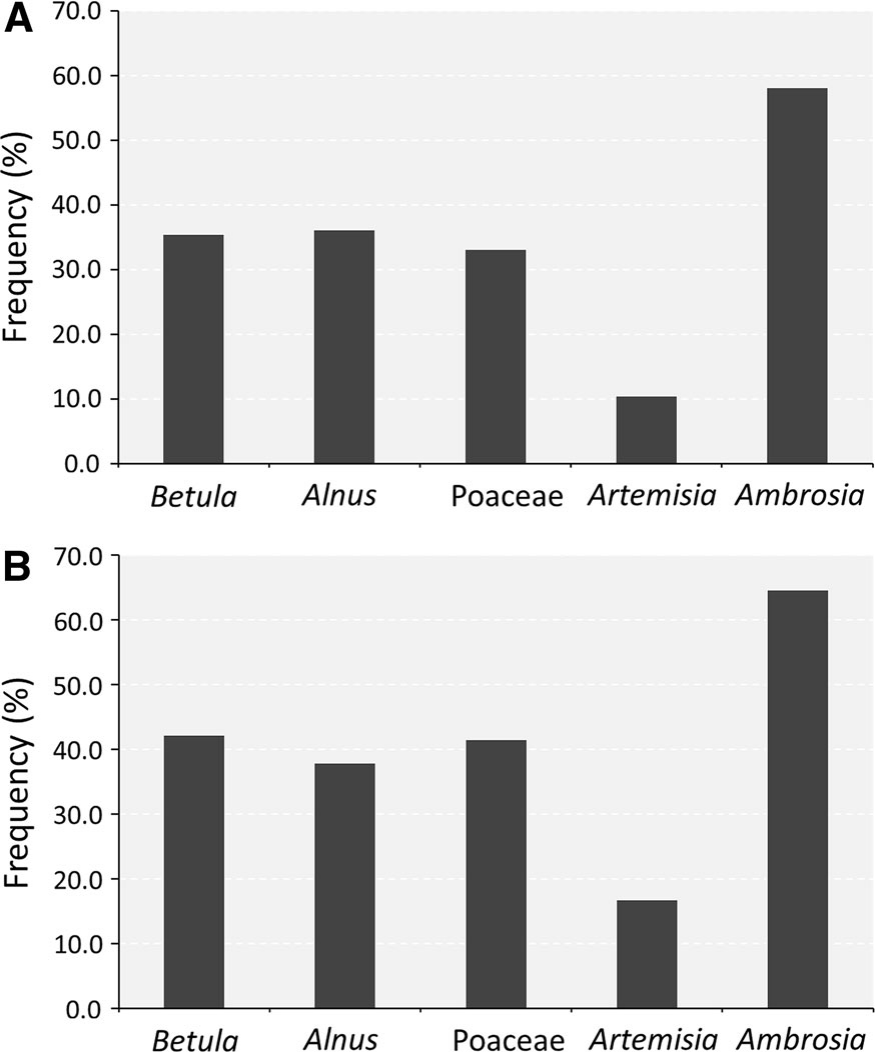
Frequency of 24-h periods with **A** mean and **B** maximum pollen levels higher at night-time (20:00–08:00)

**Fig. 2:**
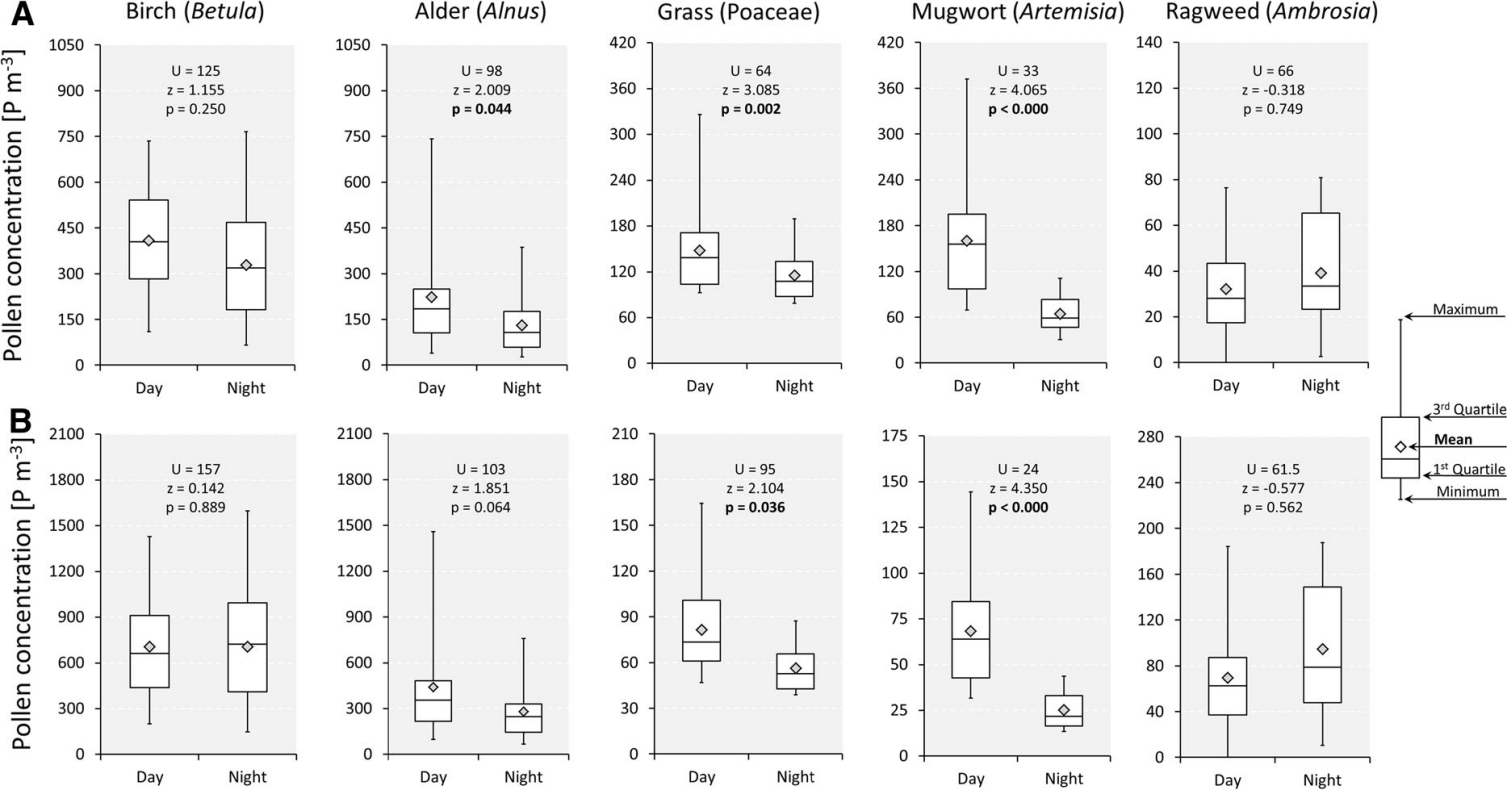
Comparison of **A** mean and **B** maximum pollen levels during day and night

Analysis of mean and maximum pollen levels recorded from 08:00 to 08:00 showed that higher atmospheric pollen concentrations were more frequently recorded during daytime hours. It was noted, however, that the frequency of higher night-time mean or maximum airborne pollen levels varied depending on pollen type, ranging from ~10.0 % for mugwort to ~35 % for grass, birch and alder, and ~60 % for ragweed (Fig. 1). The magnitude of mean and maximum concentrations of airborne pollen also varied depending on pollen type examined (Fig. 2). For the majority of pollen types investigated (i.e. alder, grass and mugwort), mean pollen levels were significantly lower at night. Differences between daytime and night-time concentrations of airborne grass and alder pollen were less distinct; the ratio between daytime and night-time levels varied from 1.3 to 1.6, respectively. On the other hand, it was found that maximum atmospheric birch pollen concentrations were almost the same at night and day (706 and 707 pollen/m^3^, respectively). Mugwort recorded very low night-time pollen concentrations; the mean and maximum *Artemisia* pollen levels were significantly lower during night than during the day (*p* < 0.000). Interestingly, for *Ambrosia*, another member of the Asteraceae family, maximum night-time pollen concentrations were over 30 % higher than recorded during the day.

Convective heat transfer, i.e. pollen laden air rising to the upper atmosphere in convection currents during daytime, is likely to be responsible for elevated pollen levels at night. This is because pollen-bearing air descends at night as it cools, thereby increasing the concentrations of pollen at ground level (Norris-Hill 1997). Other factors also need to be considered, however, such as the plant species involved and the distance that the pollen grains have to travel from the source. For instance, high concentrations of atmospheric grass pollen recorded at night might partly be the result of several grass species releasing their pollen in late evening (Peel et al. 2014). Transport from distant sources has also been indicated as a possible cause of elevated levels of birch and other pollen types at night (Skjøth et al. 2009; Fernández-Rodríguez et al. 2014). This process is particularly important for *Ambrosia* pollen that, due to the lack of local populations in Central Poland, are thought to arrive almost exclusively from distant sources, e.g. Ukraine and Hungary, and are often associated with episodes of high night-time concentrations (Smith et al. 2008; Kasprzyk et al. 2011; Šikoparija et al. 2013). It is not surprising, therefore, that levels of airborne ragweed pollen recorded in Poznań were considerably higher during night. On the other hand, mugwort pollen grains are less adopted to wind transport than other examined species and fall to the ground a short distance from the mother plant after release (Spieksma et al. 2000). This may explain why airborne mugwort pollen levels were considerable lower at night.

It should be remembered that diurnal variations in airborne pollen can be affected by several seasonal and intra-seasonal factors. As previously mentioned, the long-distance transport of atmospheric pollen can result in elevated night-time concentrations of certain pollen types. Conversely, unfavourable weather conditions, such as heavy rain that effectively washes pollen from the air, may markedly disturb the diurnal pattern of airborne pollen. For instance, Rodríguez-Rajo et al. (2010) found that rainfall causes daily average concentrations of atmospheric grass and mugwort pollen in Poznań to significantly decrease. The same authors showed that differences in local vegetation around the pollen monitoring site might also affect the intra-diurnal behaviour of pollen. However, the size of the database used in this present study (almost 2200 days of pollen data) ensures that the impact of these factors is limited and the results are robust.

In summary, this study has found that there was a considerable risk of exposure to airborne pollen at night, but this varied distinctly between pollen types. The frequency and magnitude of tree and grass pollen levels recorded during the night are comparable to the daytime values and should be considered as clinically relevant. Furthermore, areas where ragweed pollen predominantly arrives from distance sources are more likely to record higher *Ambrosia* pollen level at night than day. Physicians should be aware of this phenomenon in order to prepare better prevention strategies and allergy treatment. So dear Readers suffering from pollen allergy: Good night, sleep tight, but with windows closed, all right!
